# The Status of Rheumatoid Factor and Anti-Cyclic Citrullinated Peptide Antibody Are Not Associated with the Effect of Anti-TNFα Agent Treatment in Patients with Rheumatoid Arthritis: A Meta-Analysis

**DOI:** 10.1371/journal.pone.0089442

**Published:** 2014-02-27

**Authors:** Qianwen Lv, Yufeng Yin, Xin Li, Guangliang Shan, Xiangni Wu, Di Liang, Yongzhe Li, Xuan Zhang

**Affiliations:** 1 Department of Rheumatology & Clinical Immunology, Peking Union Medical College Hospital, Chinese Academy of Medical Sciences and Peking Union Medical College, Beijing, China; 2 Department of Epidemiology and Statistics, Institute of Basic Medical Sciences, Chinese Academy of Medical Sciences and School of Basic Medicine, Peking Union Medical College, Beijing, China; SERGAS, Santiago University Clinical Hospital, IDIS Research Laboratory 9, NEIRID Lab, Spain

## Abstract

**Objectives:**

This meta-analysis was conducted to investigate whether the status of rheumatoid factor (RF) and anti-cyclic citrullinated peptide (anti-CCP) antibody are associated with the clinical response to anti-tumor necrosis factor (TNF) alpha treatment in rheumatoid arthritis (RA).

**Methods:**

A systemic literature review was performed using the MEDLINE, SCOPUS, Cochrane Library, ISI Web of Knowledge, and Clinical Trials Register databases, and Hayden's criteria of quality assessment for prognostic studies were used to evaluate all of the studies. The correlation between the RF and anti-CCP antibody status with the treatment effect of anti-TNFα agents was analyzed separately using the Mantel Haenszel method. A fixed-effects model was used when there was no significant heterogeneity; otherwise, a random-effects model was applied. Publication bias was assessed using Egger's linear regression and a funnel plot.

**Results:**

A total of 14 studies involving 5561 RA patients meeting the inclusion criteria were included. The overall analysis showed that the pooled relative risk for the predictive effects of the RF and anti-CCP antibody status on patient response to anti-TNFα agents was 0.98 (95% CI: 0.91–1.05, p = 0.54) and 0.88 (95% CI: 0.76–1.03, p = 0.11), respectively, with I^2^ values of 43% (p = 0.05) and 67% (p<0.01), respectively. Subgroup analyses of different anti-TNFα treatments (infliximab vs. etanercept vs. adalimumab vs. golimumab), response criteria (DAS28 vs. ACR20 vs. EULAR response), follow-up period (≥6 vs. <6 months), and ethnic group did not reveal a significant association for the status of RF and anti-CCP.

**Conclusions:**

Neither the RF nor anti-CCP antibody status in RA patients is associated with a clinical response to anti-TNFα treatment.

## Introduction

Rheumatoid arthritis (RA) is a chronic inflammatory autoimmune disease that affects approximately 1% of the population worldwide [1]. OLE_LINK10Although the introduction of anti-TNFα agents has dramatically improved the outcome of RA, there unfortunately remains a proportion of RA patients who do not exhibit an adequate response to this treatment. Considering the high cost and potential side effects of anti-TNFα treatment, it is important to identify those RA patients who will be more likely to respond to these agents. Indeed, numerous studies have been conducted to investigate potential predictors for patient response to anti-TNFα therapy [2]–[4].

Both rheumatoid factor (RF) and antibodies against cyclic citrullinated peptide (anti-CCP) are regarded as serological markers of RA [5], [6]. Some studies have suggested that the status of RF or anti-CCP antibody in RA patients is associated with a clinical response to anti-TNFα treatment [7]–[14], whereas such a correlation was not found in other studies [15]–[19]. Thus, no definite conclusion has been reached to date.

We performed a meta-analysis to investigate whether RF and anti-CCP have predictive value for a clinical response to anti-TNFα treatment. Suitable studies investigating an association of the status of RF or anti-CCP and response to anti-TNFα treatment were searched and included. We also performed subgroup analyses on different variables to explore potential sources of independent predictive factors for an effect of anti-TNFα treatment.

## Methods

### Search strategy

A literature search was performed for all studies evaluating an association between the status of RF or anti-CCP antibody and a response to anti-TNFα therapy in RA patients using the Medline, Cochrane Library, SCOPUS (including EMbase), ISI Web of Knowledge, and Clinical Trials Register (clinical trials.gov) databases. The following keywords were searched: rheumatoid arthritis, anti-TNFα, rheumatoid factor, anti-cyclic citrullinated peptide antibody, clinical trials, and systematic review. Synonyms and spelling variations were taken into account (Search strategy for Scopus was listed in Table S1 in File S1). There was a limitation with regard to language, i.e., we only considered English publications, but not the year of publication. We also contacted authors to request a full-text review or specific data from studies when there was no electronic version of the full text or sufficient data for the meta-analysis. Citations were reviewed to search relevant original studies, and an electronic search alert was set to cover recent studies.

### Study selection

There were 1649 references identified by the literature search. Three individual investigators (QL, YY, & XL) evaluated the references, and the decision of inclusion was made by consensus. A study was included based on the following criteria: 1) the patients were older than 16 years old, diagnosed with RA using ACR criteria, and treated with at least one anti-TNFα agent (adalimumab, infliximab, etanercept, certolizumab, or golimumab); 2) efficacy was measured using EULAR or ACR or DAS28 criteria after a minimum duration of 12 weeks; and 3) the status of RF or anti-CCP antibody at baseline and sufficient data to calculate the risk ratio (RR) were reported in the study. The following information was extracted from each study: the study design, patients' characteristics, baseline status of RF or anti-CCP antibody, interventions, outcomes, and study duration.

### Study assessment

All of the studies included were evaluated for potential bias using Hayden's criteria of the quality assessment of prognostic studies [20] (The details of the modified criteria were provided in Table S2 in File S2). Six domains were considered in the bias assessment method: “study participation”, “study attrition”, “prognostic factor measurement”, “outcome measurement”, “confounding measurement and account”, and “analysis”. Scores of “0”, “1”, or “2” represented “high bias”, “partial bias”, and “low bias”, respectively. If the available information was insufficient to obtain a decision after contacting the authors, “unsure” was used, and no score was calculated. The total scores were calculated to show the level of study quality; studies with a score of “11–12” were rated as having relatively high quality, with “9–10” as moderate, and “less than 8” as low quality. The evaluation was performed independently by three investigators (QL, YY, & XL) to improve the validity of the results.

### Statistical analysis

We estimated the risk ratio of the RF or anti-CCP antibody status for a response to anti-TNFα treatment. The point estimate of the risk ratio (RR) and 95% confidence intervals (CI) were calculated for each study, and a Mantel Haenszel analysis was used to calculate the pooled RR and 95% CI. The heterogeneity of the effects across studies was assessed using the Chi square test and qualified by I^2^ values, which represent the proportion of between-study variability that is attributable to heterogeneity rather than to chance; I^2^ values of 25%, 50%, and 75% are referred to as low, moderate, and high heterogeneity effects, respectively. A fixed-effects model was used when there was no significant heterogeneity, whereas a random-effects model was used in other cases. Subgroup analyses were performed to investigate potential sources of heterogeneity by stratifying the different anti-TNFα agents, response criteria, duration of follow-up, and ethnic group. In addition, a sensitivity analysis was performed by the sequential omission of individual studies. Publication bias was estimated by funnel plots and an Egger's test, with p<0.05 considered as representing significant bias. All of the statistical analyses were performed using Review Manager 5.0 software, and the results of the publication bias were estimated using STATA 12.0 software.

## Results

### Characteristics of the included studies

**Figure 1:**
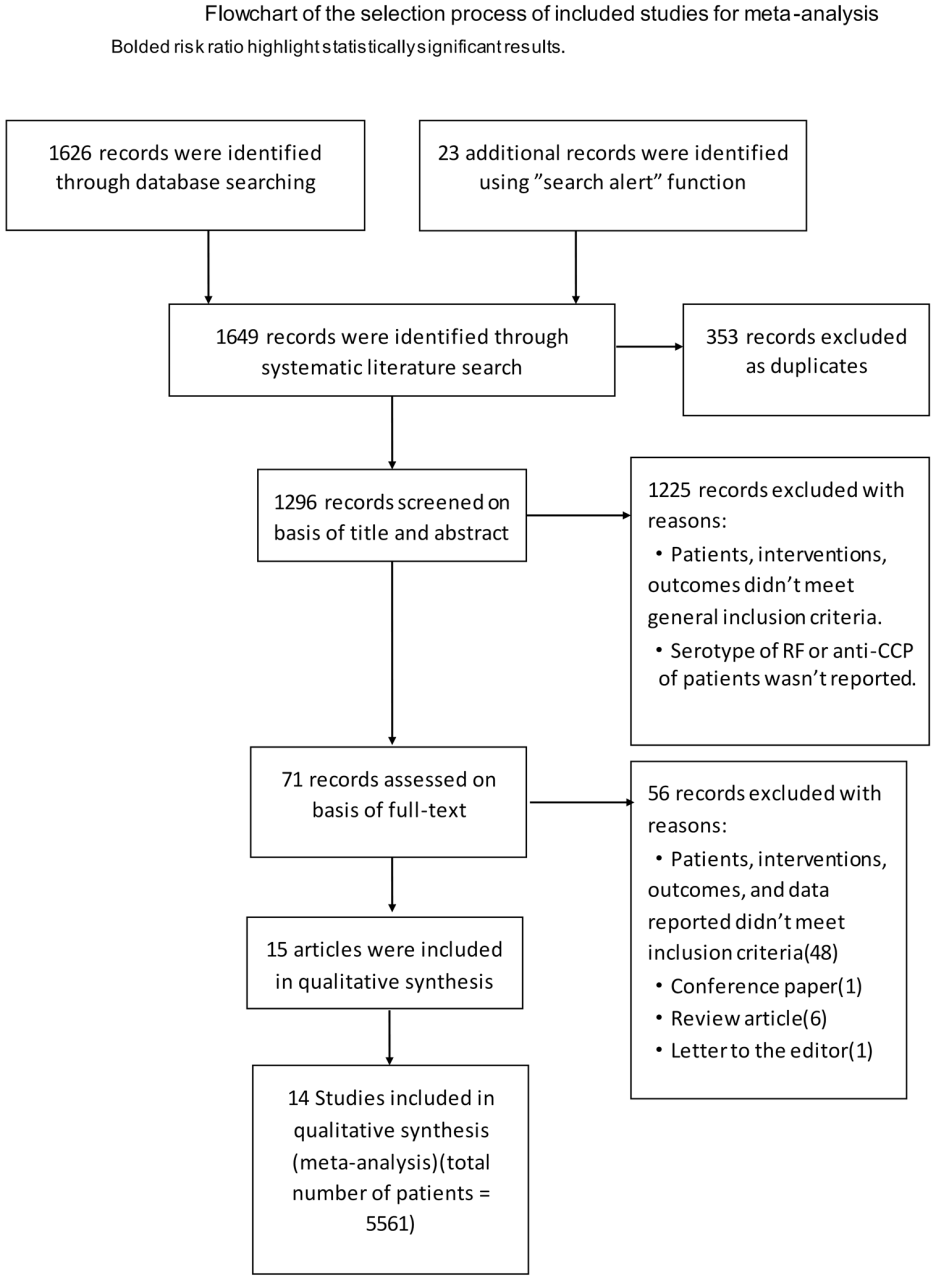
Flowchart of the study selection.

Figure 1 describes the flowchart of our literature search. According to pre-determined criteria, fifteen articles, published between 2006 and 2012, were included [7], [9]–[19], [21]–[23] (A list of articles excluded and the reasons for exclusion were presented in Table S3 in File S1). As two described the same cohort [11], [21], fourteen studies were ultimately included in this meta-analysis [7], [9], [10], [12]–[19], [21]–[23]. These studies comprised a total of 5561 RA patients: 5374 patients had their status of RF detected, and 1283 patients had their status of anti-CCP antibody detected. All but one were prospective cohort studies. All of the participants in the 14 studies had active RA, as evaluated by the number of swollen and tender joints, or DAS28 value, and the patients in all but one_ENREF_7 study had exhibited failure for at least one type of Disease-modifying Anti-Rheumatic Drug (DMARD) treatment [13]. Almost all of the patients had established RA, with a mean disease duration from 5 to 14 years. The data extracted from the 14 studies are summarized in Table 1. With regard to the anti-TNFα agents utilized, 10 studies involved infliximab, 8 involved adalimumab, 6 involved etanercept, and 1 involved golimumab.

**Table 1:** Data extracted from the included studies.

Study	Study design	No. of patients	Disease duration (yrs)	Age (yrs)	Gender (female%)	No. of swollen joints	No of tender joints	DAS28	Prior biologic used#	Intervention	Country	Primary clinical outcome (response criteria)	Study duration	Antibody measurement	Cut-off value of antibody
Braun2006	Pro*	30	12 (8.9)	50.6 (13.9)	73%	N/A	N/A	7.4 (0.95)	N/A	INF+86.7%MTX	Israel	EULAR	3.5 m	Anti-CCP: ELISA	5 IU/ml
Hyrich2006	Pro	2879	14 (9)	55 (12)	78%	12 (6)	16 (7)	6.7 (1.0)	N/A	ETA+28%MTX: 1267 pts; INF+86%MTX: 1612 pts	Britain	EULAR	6 m	N/A	N/A
Bobbio2007	Pro	126	8.3 (6.9)	57.3 (12.5)	76.5%	9.6 (5.7)	16.0 (9.9)	5.87 (0.99)	N/A	INF/ADA/ETA+CSs/NSAIDs allowed	Italy	EULAR	12 m	RF: immunonephelometry; Anti-CCP: ELISA	15 IU/ml; 5 IU/ml
Lequerre2007	Pro	76	10.5(8.6)	53.8(12.4)	81.6%	13.8(6)	10.7(7.3)	5.8(1)	N/A	INF+MTX/LEF	France	EULAR	3.5 m	RF: latex agglutination test;; Anti-CCP: ELISA	N/A
Mancarella2007	Retro	591	11.6 (7.6)	53.3 (12.7)	67%	10.4 (7.1)	17.1 (9.4)	5.9 (1.2)	N/A	INF/ETA/ADA	Italy	EULAR	6 m	N/A	20 IU/ml
Wijbrandts2007	Pro	103	10.4 (9.2)	55 (13)	69%	N/A	N/A	5.9 (1.1)	None	INF+CSs/NSAIDs allowed	The Netherlands	DAS28	4 m	N/A	N/A
Cuchacovich2008	Pro	59	11.7	48.9	87%	16.2	20	N/A	N/A	ADA+DMARDs/NSAIDs allowed	Chile	ACR20	6 m	Anti-CCP: ELISA	25 IU/ml
Wouter2008	Pro	172	8.5	53.7	79.3%	N/A	N/A	5.1	N/A	ADA+74%MTX+34%CSs	The Netherlands	EULAR	7 m	RF: ELISA; Anti-CCP: ELISA	30 IU/ml; 5 AU/ml
Alexandra2009	Pro	36	10.2 (5.8)	50.5 (10.2)	75%	7.4 (5.3)	9.5 (5)	5.2 (0.9)	None	INF+91.6%MTX	France	EULAR	12 m	RF: quantitative nephelometric test; Anti-CCP: ELISA	1000 IU/ml; 200 IU/ml
Keystone2009	Pro	179	5.3	52	80.9%	12.5	24.5	6.008	N/A	GOL+MTX	From 12 countries	ACR20	3.5 m	N/A	N/A
Potter2009	Pro	642	14 (10)	57 (11)	78%	N/A	N/A	6.7 (1)	N/A	ADA: 62 pts; ETA: 241 pts; INF: 218 pts	Britain	EULAR	6 m	RF; immunoturbidimetry; Anti-CCP: ELISA	40 U/ml; 5 U/ml
Soto2010	Pro	52	11.9	50	88.5%	16.9	21.4	5.8	N/A	ADA+stable DMARDs/NSAIDs/CSs allowed	Chile	DAS28; ACR20/50/70	6 m	RF: ELISA; Anti-CCP: ELISA	N/A; 25 IU/ml
Vasilopoulos2011	Pro	100	13.6 (6.5)	57.4 (10.8)	92%	N/A	N/A	5.6 (0.2)	N/A	INF+MTX: 24 pts; ETA±MTX: 26 pts; ADA±MTX/LEF:50 pts	Greece	DAS28	6 m	RF: immunonephelometry; Anti-CCP: ELISA	15 IU/ml; 5 U/ml
Canhao2012	Pro	516	10.4 (8.6)	50.9–54.1	88%	8 (5.4)	11.5 (7.3)	5.8 (1.2)	None	INF/ADA/ETA±DMARDs/CSs	Portugal	EULAR	6 m	RF: ELISA	IgM:5 IU/ml; IgA:20 IU/ml

*Pro, prospective clinical trial; Retro, retrospective study; N/A, not available; INF, infliximab; ADA, adalimumab; ETA, etanercept; MTX, methotrexate; LEF, leflunomide; NSAIDs, non-steroidal anti-inflammatory drugs; DMARDs, disease-modifying anti-rheumatic drugs; CSs, corticosteroids.

# Different prior biologic agents used may introduce potential heterogeneity, though the available data were insufficient for a sub-analysis.

Disease of duration, age, No. of swollen joints, No. of tender joints, DAS28 are presented as the mean (SD).

There was no study that involved patients receiving certolizumab. Only four studies investigated the status of RF, with only two investigating anti-CCP; the other eight studies measured both RF and anti-CCP. The follow-up duration of these 14 studies ranged from 14 weeks to 48 weeks.

### Study quality

Our assessment was performed on the basis of Hayden's criteria for prognostic studies [20]. The results revealed that only one of the included studies was considered to be of low quality, with a score of 7; the other thirteen studies were considered as having moderate or high quality (11 moderate and 2 high) (Table 2).

**Table 2:** The results of an assessment for bias in accordance with Hayden's criteria.

	Assessment domain						
Study	study participation	study attrition	prognostic factor measurement	outcome measurement	confounding measurement and account	analysis	total score
Braun, 2006	moderate	low	moderate	low	low	low	10
Hyrich, 2006	low	low	unsure	low	moderate	low	9*
Bobbio, 2007	low	low	low	low	high	low	10
Lequerre, 2007	low	low	moderate	low	moderate	low	10
Mancarella, 2007	low	high	moderate	low	high	low	7
Wijbrandts, 2007	low	moderate	low	low	moderate	low	10
Cuchacovich, 2008	moderate	moderate	low	low	moderate	low	10
Wouter, 2008	low	low	low	low	moderate	low	11
Alexandra, 2009	moderate	low	moderate	low	moderate	low	9
Keystone, 2009	high	low	moderate	low	low	low	9
Potter, 2009	low	moderate	low	low	moderate	low	10
Soto, 2010	moderate	low	moderate	low	moderate	low	9
Vasilopoulos, 2011	low	low	low	low	moderate	low	11
Canhao, 2012	low	moderate	moderate	low	moderate	low	9

Unsure: not enough information to evaluate.

Low, low risk of bias; moderate, moderate risk of bias; high, high risk of bias.

*One of the domains was assessed as “unsure” due to unavailable information even after the authors were contacted.

### Association evaluation of RF/anti-CCP antibody status and patient response to anti-TNFα agents

**Figure 2:**
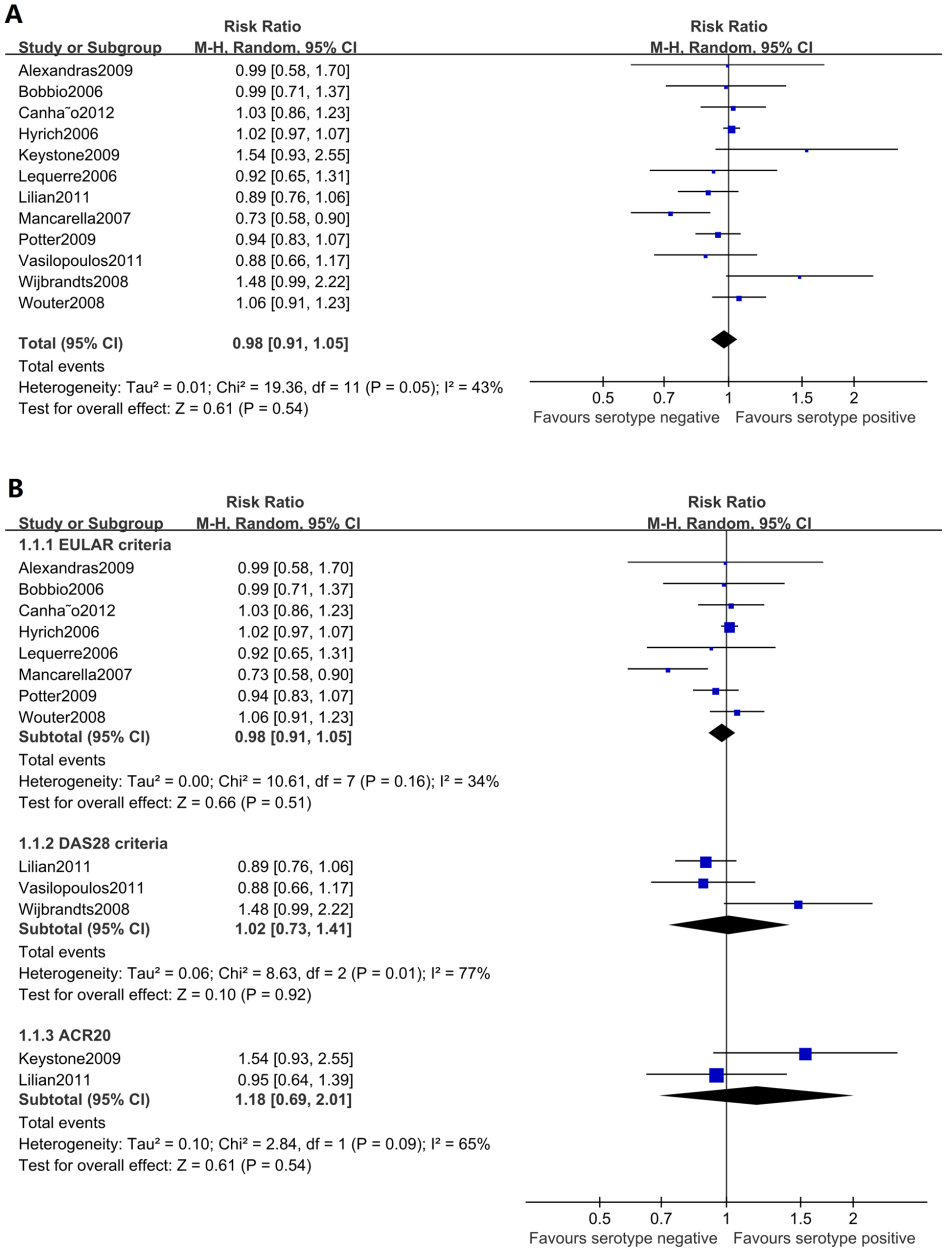
Forest graphs of the meta-analysis of RF status and response to anti-TNFα agents. The overall analysis of RF status showed a pooled RR of 0.98 (95% CI: 0.91–1.05, p = 0.54) and an I^2^ of 43%. Subgroup analyses on different response criteria revealed no significant differences.

Twelve of the included studies, with a total of 5374 patients, examined the predictive effect of the RF status for patient response to anti-TNFα agents. The overall analysis revealed that the pooled RR was 0.98 (95% CI: 0.91–1.05, p = 0.54) and that I^2^ was 43% (Figure 2a), suggesting that the RF status was not associated with a patient's response to anti-TNFα treatment, with a moderate heterogeneity observed. Subgroup analyses on the different anti-TNFα agents, follow-up periods, response criteria, and ethnic groups were performed to investigate sources of this heterogeneity, though the results revealed no significant differences (Figure 2b, Table 3).

**Table 3:** Subgroup meta-analysis of RF and RA patient response according to different anti-TNFα agents, follow-up periods, response criteria, and ethnic groups.

				Test of association			Test of heterogeneity		
Subgroup	Population	No. of studies	No. of patients	RR	95%CI	P value	Model	P value	I^2^
	overall	12	5374	0.98	0.91–1.05	0.54	R	0.05	43%
Anti-TNFα agent*	infliximab	4	1827	1.03	0.89–1.19	0.71	R	0.28	23%
	adalimumab	2	248	0.98	0.81–1.18	0.8	R	0.09	65%
	etanercept	1	1267	1.05	0.97–1.12	0.21	NA	NA	NA
	golimumab	1	178	1.54	0.93–2.55	0.09	NA	NA	NA
Follow up period	≥6 months	9	5017	0.96	0.90–1.03	0.27	R	0.13	37%
	<6 months	3	357	1.25	0.87–1.78	0.22	R	0.11	54%
Ethnic group	European	10	5146	0.98	0.91–1.06	0.59	R	0.10	39%
	South American	1	50	0.89	0.76–1.06	0.19	NA	NA	NA

R, random-effects model; NA, not applicable.

*The number of studies and number of patients receiving individual anti-TNFα agent in this table are those that we could identified after literature review and contacting the authors.

**Figure 3:**
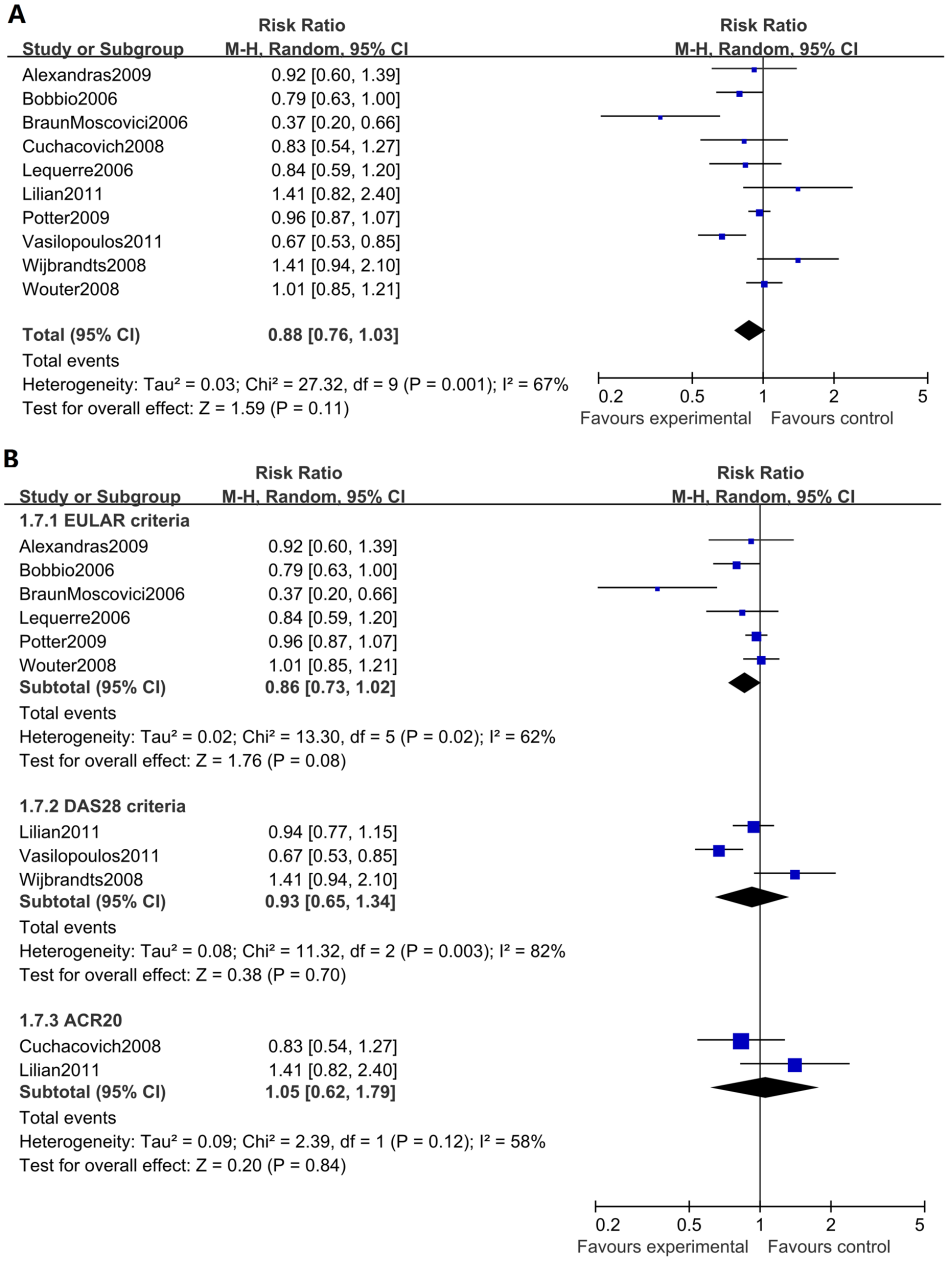
Forest graphs of the meta-analysis of anti-CCP antibody status and response to anti-TNFα agents. The overall analysis of anti-CCP antibody status showed a pooled RR of 0.88 (95% CI: 0.76–1.03, p = 0.11) and an I^2^ of 67%. Subgroup analyses on different response criteria revealed no significant differences.

Ten studies, with a total of 1283 RA patients, were analyzed for an association of anti-CCP antibody status and patient response to anti-TNFα treatment. The overall meta-analysis showed no association between the status of anti-CCP antibody and a patient's response to anti-TNFα treatment, with a pooled RR of 0.88 (95% CI: 0.76–1.03, p = 0.11) and an I^2^ of 67% (Figure 3a). When we performed a subgroup analysis to explore potential heterogeneity, we did not identify any association by stratifying the studies using either different anti-TNFα agents, follow-up periods, response criteria, or ethnic groups (Figure 3b, Table 4).

**Table 4:** Subgroup meta-analysis of anti-CCP and RA patient response according to different anti-TNFα agents, follow-up periods, response criteria, and ethnic groups.

				Test of association			Test of heterogeneity		
Subgroup	Population	No. of studies	No. of patients	RR	95%CI	P value	Model	P value	I^2^
	overall	10	1283	0.88	0.76–1.03	0.11	R	<0.01	67%
Anti-TNFα agent*	infliximab	5	345	0.79	0.55–1.13	0.19	R	<0.01	78%
	adalimumab	3	297	1.01	0.88–1.17	0.86	F	0.69	0%
Follow up period	≥6 months	7	1074	0.90	0.80–1.01	0.06	R	0.09	46%
	<6 months	3	209	0.78	0.40–1.53	0.47	R	<0.01	86%
Ethnic group	European	7	1144	0.90	0.79–1.04	0.17	R	0.02	60%
	South American	2	109	1.01	0.79–1.30	0.91	F	0.38	0
	Asian	1	30	0.37	0.20–0.66	<0.05	NA	NA	NA

F, fixed-effects model; R, random-effects model; NA, not applicable.

*The number of studies and number of patients receiving individual anti-TNFα agent in this table are those that we could identified after literature review and contacting the authors.

### Sensitivity and publication bias

**Figure 4:**
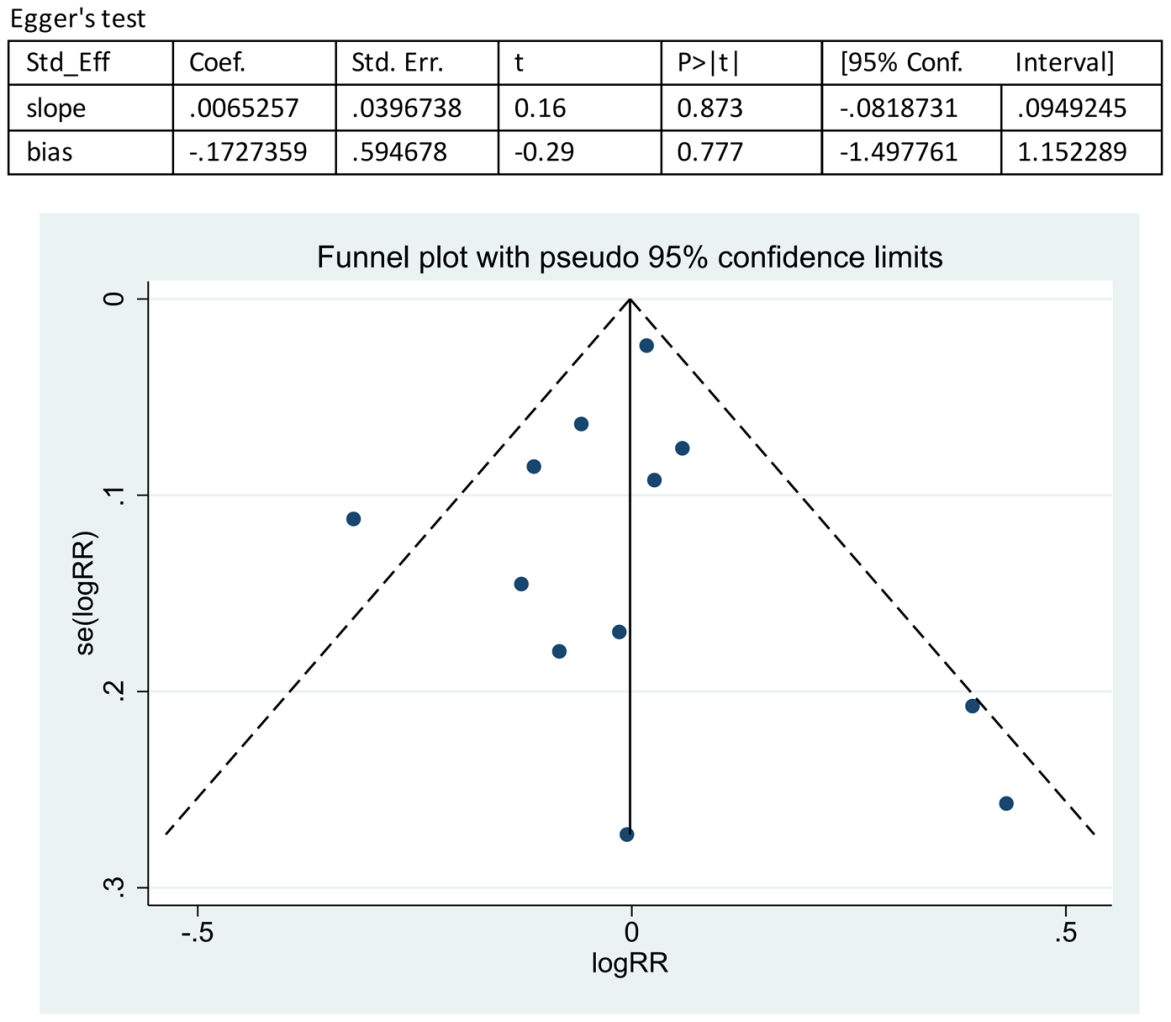
Overall analysis of publication bias on the effect of RF status on the response to anti-TNFα treatment. Egger's linear regression test was performed to quantify publication bias. The p values of the RF status analysis were 0.777. The funnel plot showed no significant evidence of asymmetry.

**Figure 5:**
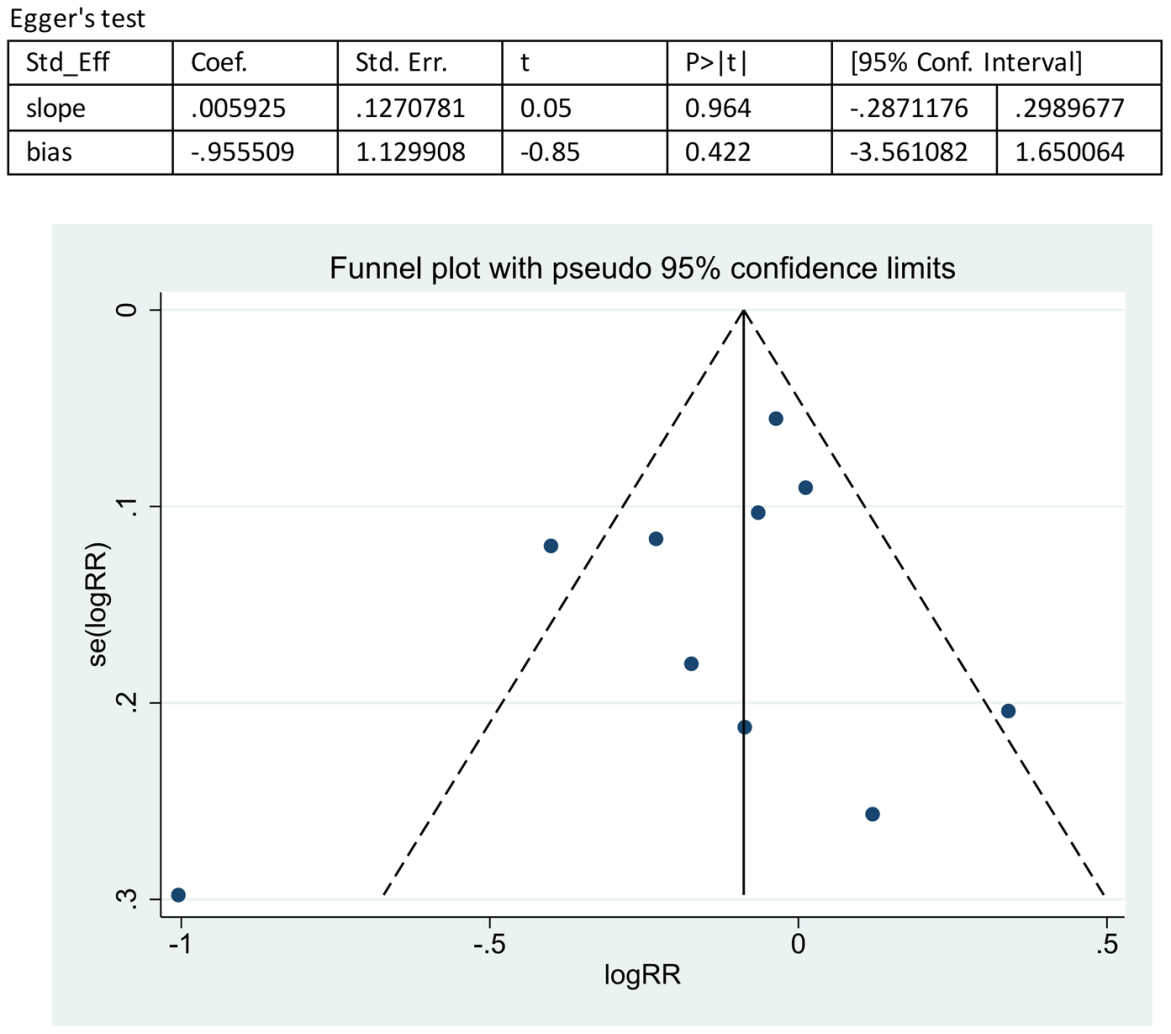
Overall analysis of publication bias on the effect of anti-CCP antibody status on the response to anti-TNFα treatment. Egger's linear regression test was performed to quantify publication bias. The p values of the anti-CCP antibody status analysis were 0.422. The funnel plot showed no significant evidence of asymmetry.

Sensitivity analyses were performed based on the results of the bias assessments, Hayden's criteria of quality assessment for prognostic studies, by the sequential omission of individual studies, and the result revealed that the significance estimate of the overall pooled RR was not influenced by omitting any single study. The publication bias was evaluated by a funnel plot, which showed no significant evidence of asymmetry (Figures 4–5). We also performed an Egger's linear regression test to quantify the publication bias. The p values of the RF and anti-CCP antibody status analyses were 0.777 and 0.422 (Figures 4–5), and all other Egger's test p values in this meta-analysis were >0.05, suggesting no significant bias in the analysis.

## Discussion

Previous single studies have suggested that the presence of RF and anti-CCP antibody is associated with a reduced response to anti-TNFα treatment [7]–[14];_ENREF_9 however, a number of studies have reported a contrasting conclusion [15]–[19]. Thus, to investigate whether the status of RF or anti-CCP antibody in RA patients has predictive value for a clinical response to anti-TNFα treatment, we performed a meta-analysis by a comprehensive literature search and inclusion of all of the available qualifying studies. The results of our meta-analysis demonstrated that there was no association between the status of RF and patient response to anti-TNFα treatment. Furthermore, the status of anti-CCP antibody was also not associated with patient response, with a pooled RR of 0.88 and 95% CI of 0.76–1.03 (p = 0.11), indicating a tendency of association between the absence of anti-CCP antibodies and a patient's response to anti-TNFα therapy. Therefore the cumulative results did not support or completely reject the association between the presence of RF and/or anti-CCP and the effect of treatment with TNFα inhibitors. Anti-CCP is an independent predictor of radiological damage and progression in RA patients [24], [25], and anti-CCP-positive patients have a relatively worse prognosis. As a result, patients with a different anti-CCP antibody status may have a different baseline of the disease, which could influence their response to anti-TNFα agents. To explore this influence, analyses should be performed to investigate potential differences in the baseline characteristics between groups stratified by the status of anti-CCP antibody. Alternatively, only analyses based on studies with placebo groups are powered to definitively identify such an association. Unfortunately, such information was not available for the included studies.

This meta-analysis does have some limitations. First, only 14 studies were included in this meta-analysis, with 12 studies reporting the status of RF and 10 studies reporting the status of anti-CCP antibody. Nonetheless, after a comprehensive literature search covering five databases and the selection of eligible studies by three different investigator strictly according to the inclusion criteria, most of the included studies were assessed with moderate-to-high quality. Therefore, it is reasonable to obtain a conclusion based on the studies included in this meta-analysis. However, as potential heterogeneity exists between studies after subgroup analyses were performed, the result of our analyses should be interpreted with caution, and our conclusions should be updated using new, larger studies in the future. Secondly, all of the patients in the studies included in our meta-analysis had long-standing disease, with a mean value of disease duration ranging from 5 to 14 years, and had previously shown failure with at least one type of DMARD. Indeed, patients with more severe chronic disease as a result of irreversible joint damage may be less likely to respond to anti-TNFα treatment [17]. Thirdly, most of the included studies reported a combined treatment of DMARDs in a proportion of the patients enrolled. Because studies have demonstrated improved outcomes among patients receiving combination therapy with MTX plus anti-TNFα [17], the heterogeneity of combined therapy among the studies may have introduced potential bias.

In summary, our meta-analysis indicated that the status of RF and anti-CCP are not associated with the clinical response to anti-TNFα treatment in RA patients.

## Supporting Information

File S1 **File includes Tables S1–S3.** Table S1: Search strategy for Scopus. Table S2: Guidelines for assessing quality in prognostic studies. Table S3: A list of the article not included in the study (and the reasons).
(DOCX)

File S2 **PRISMA Checklist.**
(DOC)

## References

[1] Harris ED (1990) Rheumatoid Arthritis. New England Journal of Medicine 322: 1277–1289. 2271017 10.1056/NEJM199005033221805

[2] Radovits BJ, Kievit W, Fransen J, van de Laar MA, Jansen TL, et al (2009) Influence of age on the outcome of antitumour necrosis factor alpha therapy in rheumatoid arthritis. Ann Rheum Dis 68: 1470–1473. 19015210 10.1136/ard.2008.094730

[3] Kristensen LE, Kapetanovic MC, Gulfe A, Soderlin M, Saxne T, et al (2008) Predictors of response to anti-TNF therapy according to ACR and EULAR criteria in patients with established RA: results from the South Swedish Arthritis Treatment Group Register. Rheumatology (Oxford) 47: 495–499. 18316338 10.1093/rheumatology/ken002

[4] Gibbons LJ, Hyrich KL (2009) Biologic therapy for rheumatoid arthritis: clinical efficacy and predictors of response. BioDrugs 23: 111–124. 19489652 10.2165/00063030-200923020-00004

[5] Pruijn GJ, Wiik A, van Venrooij WJ (2010) The use of citrullinated peptides and proteins for the diagnosis of rheumatoid arthritis. Arthritis Research & Therapy 12: 203. 20236483 10.1186/ar2903 PMC2875630

[6] Avouac J, Gossec L, Dougados M (2006) Diagnostic and predictive value of anti-cyclic citrullinated protein antibodies in rheumatoid arthritis: a systematic literature review. Ann Rheum Dis 65: 845–851. 16606649 10.1136/ard.2006.051391 PMC1798205

[7] Vasilopoulos Y, Bagiatis V, Stamatopoulou D, Zisopoulos D, Alexiou I, et al (2011) Association of anti-CCP positivity and carriage of TNFRII susceptibility variant with anti-TNF-alpha response in rheumatoid arthritis. Clinical & Experimental Rheumatology 29: 701–704. 21813066

[8] Tanaka Y, Takeuchi T, Inoue E, Saito K, Sekiguchi N, et al (2008) Retrospective clinical study on the notable efficacy and related factors of infliximab therapy in a rheumatoid arthritis management group in Japan: one-year clinical outcomes (RECONFIRM-2). Modern Rheumatology 18: 146–152. 18283523 10.1007/s10165-008-0026-3 PMC2279153

[9] Potter C, Hyrich KL, Tracey A, Lunt M, Plant D, et al (2009) Association of rheumatoid factor and anti-cyclic citrullinated peptide positivity, but not carriage of shared epitope or PTPN22 susceptibility variants, with anti-tumour necrosis factor response in rheumatoid arthritis. Ann Rheum Dis 68: 69–74. 18375541 10.1136/ard.2007.084715 PMC2596303

[10] Mancarella L, Bobbio-Pallavicini F, Ceccarelli F, Falappone PC, Ferrante A, et al (2007) Good clinical response, remission, and predictors of remission in rheumatoid arthritis patients treated with tumor necrosis factor-alpha blockers: the GISEA study.[Erratum appears in J Rheumatol. 2007 Sep;34(9):1947]. Journal of Rheumatology 34: 1670–1673. 17611987

[11] Klaasen R, Cantaert T, Wijbrandts CA, Teitsma C, Gerlag DM, et al (2011) The value of rheumatoid factor and anti-citrullinated protein antibodies as predictors of response to infliximab in rheumatoid arthritis: an exploratory study. Rheumatology 50: 1487–1493. 21454308 10.1093/rheumatology/ker010

[12] Cuchacovich M, Catalan D, Wainstein E, Gatica H, Soto L, et al (2008) Basal anti-cyclic citrullinated peptide (anti-CCP) antibody levels and a decrease in anti-CCP titres are associated with clinical response to adalimumab in rheumatoid arthritis. Clinical & Experimental Rheumatology 26: 1067–1073. 19210871

[13] Canhao H, Rodrigues AM, Mourao AF, Martins F, Santos MJ, et al (2012) Comparative effectiveness and predictors of response to tumour necrosis factor inhibitor therapies in rheumatoid arthritis. Rheumatology (Oxford) 51: 2020–2026. 22843791 10.1093/rheumatology/kes184 PMC3475979

[14] Braun-Moscovici Y, Markovits D, Zinder O, Schapira D, Rozin A, et al (2006) Anti-cyclic citrullinated protein antibodies as a predictor of response to anti-tumor necrosis factor-alpha therapy in patients with rheumatoid arthritis. Journal of Rheumatology 33: 497–500. 16511906

[15] Soto L, Sabugo F, Catalan D, Wurmann P, Cermenatti T, et al (2011) The presence of anti-citrullinated protein antibodies (ACPA) does not affect the clinical response to adalimumab in a group of RA patients with the tumor necrosis factor (TNF) alpha-308[NON-BREAKING SPACE]G/G promoter polymorphism. Clinical Rheumatology 30: 391–395. 21234628 10.1007/s10067-011-1679-4

[16] Lequerre T, Jouen F, Brazier M, Clayssens S, Klemmer N, et al (2007) Autoantibodies, metalloproteinases and bone markers in rheumatoid arthritis patients are unable to predict their responses to infliximab. Rheumatology 46: 446–453. 16899502 10.1093/rheumatology/kel262

[17] Hyrich KL, Watson KD, Silman AJ, Symmons DPM (2006) British Society for Rheumatology Biologics R (2006) Predictors of response to anti-TNF-alpha therapy among patients with rheumatoid arthritis: results from the British Society for Rheumatology Biologics Register. Rheumatology 45: 1558–1565. 16705046 10.1093/rheumatology/kel149

[18] Bobbio-Pallavicini F, Caporali R, Alpini C, Avalle S, Epis OM, et al (2007) High IgA rheumatoid factor levels are associated with poor clinical response to tumour necrosis factor alpha inhibitors in rheumatoid arthritis. Annals of the Rheumatic Diseases 66: 302–307. 17079248 10.1136/ard.2006.060608 PMC1856018

[19] Alexandra, Nicaise-Roland P, Hayem G, Palazzo E, Dieude P, et al (2009) Prospective cohort study of effects of infliximab on rheumatoid factor, anti-cyclic citrullinated peptide antibodies and antinuclear antibodies in patients with long-standing rheumatoid arthritis. Joint, Bone, Spine: Revue du Rhumatisme 76: 248–253. 10.1016/j.jbspin.2008.09.010 19208451

[20] Hayden JA, ^te' PC, Bombardier C (2006) Evaluation of the Quality of Prognosis Studies in Systematic Reviews. Ann Intern Med 144: 427–437. 16549855 10.7326/0003-4819-144-6-200603210-00010

[21] Wijbrandts CA, Dijkgraaf MG, Kraan MC, Vinkenoog M, Smeets TJ, et al (2008) The clinical response to infliximab in rheumatoid arthritis is in part dependent on pretreatment tumour necrosis factor alpha expression in the synovium. Ann Rheum Dis 67: 1139–1144. 18055470 10.1136/ard.2007.080440 PMC2564801

[22] Bos WH, Bartelds GM, Wolbink GJ, de Koning MHMT, van de Stadt RJ, et al (2008) Differential response of the rheumatoid factor and anticitrullinated protein antibodies during adalimumab treatment in patients with rheumatoid arthritis. Journal of Rheumatology 35: 1972–1977. 18785316

[23] Keystone EC, Genovese MC, Klareskog L, Hsia EC, Hall ST, et al. (2009) Golimumab, a human antibody to tumour necrosis factor {alpha} given by monthly subcutaneous injections, in active rheumatoid arthritis despite methotrexate therapy: the GO-FORWARD Study. Annals of the Rheumatic Diseases. pp. 789–796. 10.1136/ard.2008.099010 PMC2674549 19066176

[24] Forslind K, Ahlmen M, Eberhardt K, Hafstrom I, Svensson B (2004) Prediction of radiological outcome in early rheumatoid arthritis in clinical practice: role of antibodies to citrullinated peptides (anti-CCP). Ann Rheum Dis 63: 1090–1095. 15308518 10.1136/ard.2003.014233 PMC1755129

[25] Bongi SM, Manetti R, Melchiorre D, Turchini S, Boccaccini P, et al (2004) Anti-cyclic citrullinated peptide antibodies are highly associated with severe bone lesions in rheumatoid arthritis anti-CCP and bone damage in RA. Autoimmunity 37: 495–501. 15621577 10.1080/08916930400011965

