# Supplementary material for: The Status of Rheumatoid Factor and Anti-Cyclic Citrullinated Peptide Antibody Are Not Associated with the Effect of Anti-TNFα Agent Treatment in Patients with Rheumatoid Arthritis: A Meta-Analysis
Source: PLoS One. 2014 Feb 27;9(2):e89442. doi: 10.1371/journal.pone.0089442 (PMC3937352; doi:10.1371/journal.pone.0089442)
Supplement: File S1 — File includes Tables S1–S3. Table S1: Search strategy for Scopus. Table S2: Guidelines for assessing quality in prognostic studies. Table S3: A list of the article not included in the study (and the reasons). (DOCX) [file pone.0089442.s001.docx]

Table S1. Search strategy for Scopus

| # ID | Search term |
| --- | --- |
| 1 | (rheumatoid adj1 arthritis).mp. |
| 2 | tnf*.mp. |
| 3 | tumo?r necrosis factor*.mp. |
| 4 | antitnf*.mp. |
| 5 | anti-tnf*.mp. |
| 6 | antitumo?r necrosis factor*.mp. |
| 7 | anti-tumo?r necrosis factor*.mp. |
| 8 | (infliximab* or remicade* or cA2*).mp. |
| 9 | (etanercept* or enbrel* or p75TNFR-Fc*).mp |
| 10 | (adalimumab* or humira* or D2E7*).mp. |
| 11 | (certolizumab* or cimzia* or CDP870*).mp. |
| 12 | (golimumab* or simponi* or CNTO-148*).mp. |
| 13 | 2 or 3 or 4 or 5 or 6 or 7 or 8 or 9 or 10 or 11 or 12 |
| 14 | (rheumatoid factor or RF).mp. |
| 15 | (anti-citrullinated peptide antibody or anticitrullinated peptide antibody or ACPA).mp. |
| 16 | (anti-cyclic citrullinated peptide or anticyclic citrullinated peptide or anti-CCP).mp. |
| 17 | (anti-citrullinated vimentin antibody or anti-modifed citrullinated vimentin or anti-mutated citrullinated vimentin or anti-Sa or anti-MCV).mp. |
| 18 | 14 or 15 or 16 or 17 |
| 21 | (clinical trial).mp |
| 22 | (meta adj1 analy*).mp. |
| 23 | metaanaly*.mp. |
| 24 | (systematic* adj3 (review* or overview* or litera* or search*)).mp |
| 25 | 21or22 or 23 or 24 |
| 27 | 1 and 13 and 18 and 25 |

Table S2. Guidelines for assessing quality in prognostic studies.

| **Potential Bias** | **Items to be considered for assessment of potential opportunity for bias** |
| --- | --- |
| **Study participation**  The study sample represents the population of interest on key characteristics, sufficient to limit potential bias to the results.  Yes*  Partly  No  Unsure | The source population or population of interest is adequately described for key characteristics.  The sampling frame and recruitment are adequately described(e.g., including period and place of recruitment).  Inclusion and exclusion criteria are adequately described.  There is adequate participation in the study by eligible individuals(which is evaluated by the relative size of the study).  The baseline study sample (i.e., individuals entering the study) is adequately described for key characteristics. |
| **Study attrition**  Loss to follow-up (from sample to study population) is not associated with key characteristics (i.e., the study data adequately represent the sample), sufficient to limit potential bias.  Yes  Partly  No  Unsure | Response rate (i.e., proportion of study sample completing the study and providing outcome data) is adequate.  Attempts to collect information on participants who dropped out of the study are described.  Reasons for loss to follow-up are provided.  Participants lost to follow-up are adequately described for key characteristics.  There are no important differences between key characteristics and outcomes in participants who completed the study and those who did not. |
| **Prognostic factor measurement**  The prognostic factor of interest is adequately measured in study participants to sufficiently limit potential bias.  Yes  Partly  No  Unsure | A clear definition or description of the prognostic factor measured is provided (as RF and anti-CCP are the prognostic factors in these studies, the level of antibody, the method of measurement are needed to limit potential bias).  Continuous variables(e.g., RF and anti-CCP) are reported or appropriate cut-points are used.  The prognostic factor measure and method are adequately valid and reliable to limit misclassification bias.  Adequate proportion of the study sample has complete data for prognostic factors.  The method and setting of measurement are the same for all study participants.  Appropriate methods are used if imputation is used for missing prognostic factor data. |
| **Outcome measurement**  The outcome of interest is adequately measured in study participants to sufficiently limit potential bias.  Yes  Partly  No  Unsure | A clear definition of the outcome of interest is provided, including duration of follow-up(e.g., defining the clinical response as the improve of DAS28 is larger than 3.2 after treatment of 12 weeks).  The measurement and calculation of outcome are well defined(e.g., the EULAR response criteria, DAS28 criteria and ACR 20/50/70 criteria are applied which have clear definition) and carried out reliably by trained investigators(e.g., the evaluation of pain score assessment or swollen joint count are performed by physicians).  The method and setting of measurement are the same for all study participants. |
| **Confounding measurement and account**  Important potential confounders are appropriately accounted for, limiting potential bias with respect to the prognostic factor of interest.  Yes  Partly  No  Unsure | Almost all important confounders, including treatments, are measured.  Clear definitions of the important confounders measured are provided (e.g., including dose, level, and duration of exposures).  Measurement of all important confounders is adequately valid and reliable (e.g., may include relevant outside sources of information on measurement properties, also characteristics, such as blind measurement and limited reliance on recall).  The method and setting of confounding measurement are the same for all study participants.  Appropriate methods are used if imputation is used for missing confounder data.  Important potential confounders are accounted for in the study design (e.g., matching for key variables, stratification, or initial assembly of comparable groups).  Important potential confounders are accounted for in the analysis (i.e., appropriate adjustment). |
| **Analysis**  The statistical analysis is appropriate for the design of the study, limiting potential for presentation of invalid results.  Yes  Partly  No  Unsure | There is sufficient presentation of data to assess the adequacy of the analysis(e.g., including additional data presented by researchers).  The strategy for model building (i.e., inclusion of variables) is appropriate and is based on a conceptual framework or model.  The selected model is adequate for the design of the study.  The original results are available and there is no selective reporting of results. |

*Yes means low risk of potential bias; Partly means moderate risk of potential bias; No means high risk of potential bias.

Table S3. A list of the article not included in the study (and the reasons)

| No. | Studies | Reasons for exclusion | The Criteria which was not met* | Number of studies |
| --- | --- | --- | --- | --- |
| 1 | Quartuccio 2009[[1](#_ENREF_1)], Reneses 2009[[2](#_ENREF_2)], Schiff 2009[[3](#_ENREF_3)], Secchiero 2010[[4](#_ENREF_4)], Van Tuyl 2010[[5](#_ENREF_5)] | The participates were not treated with anti-TNF agents. | No.1 | 5 |
| 2 | Avouac 2006[[6](#_ENREF_6)] | A systematic literature with no TNFi treatment as the intervention. | No.1 | 1 |
| 3 | De Rycke 2005[[7](#_ENREF_7)], Braun-Moscovici 2008 [[8](#_ENREF_8)], De Vries Bouwstra 2008[[9](#_ENREF_9)], Vastesaeger 2009[[10](#_ENREF_10)], Nozaki 2010[[11](#_ENREF_11)], Dirven 2012[[12](#_ENREF_12)] | Efficacy was measured by percentage change in CRP and ESR instead of EULAR/ACR/DAS28. | No.2 | 6 |
| 4 | Ceccarelli 2011[[13](#_ENREF_13)] | The outcome of EULAR/ACR/DAS28 criterias were not compared between RF or anti-CCP subgroups. | No.2 | 1 |
| 5 | Atzeni 2006[[14](#_ENREF_14)], Ahmed 2006[[15](#_ENREF_15)], Morozzi 2007 [[16](#_ENREF_16)], Klaasen 2009[[17](#_ENREF_17)], Bos WH 2009[[18](#_ENREF_18)], Dejaco 2010[[19](#_ENREF_19)], Mu Rong 2010[[20](#_ENREF_20)], Onishi Sachiko 2010[[21](#_ENREF_21)], Charles 2000[[22](#_ENREF_22)], Alessandri 2004[[23](#_ENREF_23)], Criswell 2004[[24](#_ENREF_24)], Nissinen 2004[[25](#_ENREF_25)], Argyropoulou 2005[[26](#_ENREF_26)], Voulgari 2005[[27](#_ENREF_27)], Allaart 2006[[28](#_ENREF_28)], Parker 2007 [[29](#_ENREF_29)], Scali 2007 [[30](#_ENREF_30)], Yamanaka 2007[[31](#_ENREF_31)], Tanaka 2008[[32](#_ENREF_32)], Canete 2009[[33](#_ENREF_33)], Mattey 2009[[34](#_ENREF_34)], Canete 2011[[35](#_ENREF_35)], Di Muzio 2011[[36](#_ENREF_36)], Klarenbeek 2011[[37](#_ENREF_37)], Curtis 2012[[38](#_ENREF_38)], Van Eijk 2012[[39](#_ENREF_39)] | It didn’t report the number of responders and non-responders to TNFi treatment in neither RF nor anti-CCP positive/negative groups at baseline, in which case we can’t calculate the risk ratio. | No.3 | 26 |
| 6 | Cavazzana 2007[[40](#_ENREF_40)], Stojanovic 2011[[41](#_ENREF_41)], Wang 2011[[42](#_ENREF_42)], Takeuchi 2011[[43](#_ENREF_43)] | No RF or anti-CCP at baseline were reported. | No.3 | 4 |
| 7 | Van Vollenhoven 2012[[44](#_ENREF_44)], Zivojinovic 2012[[45](#_ENREF_45)] | It didn’t compare the responders between antibody positive group and negative group. As a result, the number of responders and non-responders to TNFi treatment in neither antibody positive/negative groups at baseline weren’t reported. | No.3 | 2 |
| 8 | Lagana 2009[[46](#_ENREF_46)] | Not only clinical response criteria didn’t meet the inclusion criteria, but also the number of responders and non-responders in RF positive or negative groups was reported. | No.2 and No.3 | 1 |
| 9 | Van der Kooij 2009[[47](#_ENREF_47)] | The anti-TNF agent was not continuously used to treat the participates in this study. And It didn’t report the number of responders and non-responders to TNFi treatment in anti-CCP positive or negative groups at baseline. | No.1 and No.3 | 1 |
| 10 | Verstappen 2011[[48](#_ENREF_48)] | Only part of the participates were diagnosed as RA. And it didn’t report the number of responders and non-responders to TNFi treatment in neither RF nor anti-CCP positive/negative groups at baseline. | No.1 and No.3 | 1 |

* Inclusion criteria: No.1 The patients were older than 16 years old, diagnosed with RA using ACR criteria, and treated with at least one anti-TNFα agent (adalimumab, infliximab, etanercept, certolizumab, and golimumab); No.2 Efficacy was measured with EULAR or ACR or DAS28 criteria after a minimum duration of 12 weeks; and No.3 The serotype of RF or anti-CCP antibody at baseline and sufficient data to calculate the risk ratio (RR) were reported in the study.

**REFERENCES**

1. Quartuccio L, Fabris M, Salvin S, Atzeni F, Saracco M, et al. (2009) Rheumatoid factor positivity rather than anti-CCP positivity, a lower disability and a lower number of anti-TNF agents failed are associated with response to rituximab in rheumatoid arthritis. Rheumatology 48: 1557-1559.

2. Reneses S, Gonzalez-Escribano MF, Fernandez-Suarez A, Pestana L, Davila B, et al. (2009) The value of HLA-DRB1 shared epitope, -308 tumor necrosis factor-alpha gene promoter polymorphism, rheumatoid factor, anti-citrullinated peptide antibodies, and early erosions for predicting radiological outcome in recent-onset rheumatoid arthritis. Journal of Rheumatology 36: 1143-1149.

3. Schiff M, Pritchard C, Huffstutter JE, Rodriguez-Valverde V, Durez P, et al. (2009) The 6-month safety and efficacy of abatacept in patients with rheumatoid arthritis who underwent a washout after anti-tumour necrosis factor therapy or were directly switched to abatacept: the ARRIVE trial. Annals of the Rheumatic Diseases. pp. 1708-1714.

4. Secchiero P, Corallini F, Castellino G, Bortoluzzi A, Caruso L, et al. (2010) Baseline serum concentrations of TRAIL in early rheumatoid arthritis: relationship with response to disease-modifying antirheumatic drugs. Journal of Rheumatology 37: 1461-1466.

5. van Tuyl LHD, Voskuyl AE, Boers M, Geusens P, Landewe RBM, et al. (2010) Baseline RANKL:OPG ratio and markers of bone and cartilage degradation predict annual radiological progression over 11 years in rheumatoid arthritis. Annals of the Rheumatic Diseases 69: 1623-1628.

6. Avouac J, Gossec L, Dougados M (2006) Diagnostic and predictive value of anti-cyclic citrullinated protein antibodies in rheumatoid arthritis: a systematic literature review. Ann Rheum Dis 65: 845-851.

7. De Rycke L, Verhelst X, Kruithof E, Van den Bosch F, Hoffman IEA, et al. (2005) Rheumatoid factor, but not anti-cyclic citrullinated peptide antibodies, is modulated by infliximab treatment in rheumatoid arthritis. Annals of the Rheumatic Diseases 64: 299-302.

8. Braun-Moscovici Y, Markovits D, Rozin A, Toledano K, Nahir AM, et al. (2008) Anti-tumor necrosis factor therapy: 6 year experience of a single center in northern Israel and possible impact of health policy on results. Israel Medical Association Journal: Imaj 10: 277-281.

9. de Vries-Bouwstra JK, Goekoop-Ruiterman YPM, Verpoort KN, Schreuder GMT, Ewals JAPM, et al. (2008) Progression of joint damage in early rheumatoid arthritis: association with HLA-DRB1, rheumatoid factor, and anti-citrullinated protein antibodies in relation to different treatment strategies. Arthritis & Rheumatism 58: 1293-1298.

10. Vastesaeger N, Xu S, Aletaha D, St Clair EW, Smolen JS (2009) A pilot risk model for the prediction of rapid radiographic progression in rheumatoid arthritis. Rheumatology 48: 1114-1121.

11. Nozaki Y, Nagare Y, Hino S, Yano T, Kishimoto K, et al. (2010) Therapeutic strategy and significance of serum rheumatoid factor in patients with rheumatoid arthritis during infliximab treatment. Nihon Rinsho Meneki Gakkai Kaishi 33: 135-141.

12. Dirven L, Visser K, Klarenbeek NB, Ewals JAPM, Han KH, et al. (2012) Towards personalized treatment: predictors of short-term HAQ response in recent-onset active rheumatoid arthritis are different from predictors of rapid radiological progression. Scandinavian Journal of Rheumatology 41: 15-19.

13. Ceccarelli F, Perricone C, Fabris M, Alessandri C, Iagnocco A, et al. (2011) Transforming growth factor beta 869C/T and interleukin 6 -174G/C polymorphisms relate to the severity and progression of bone-erosive damage detected by ultrasound in rheumatoid arthritis. Arthritis Research & Therapy 13: R111.

14. Atzeni F, Sarzi-Puttini P, Dell' Acqua D, de Portu S, Cecchini G, et al. (2006) Adalimumab clinical efficacy is associated with rheumatoid factor and anti-cyclic citrullinated peptide antibody titer reduction: a one-year prospective study. Arthritis Research & Therapy 8: R3.

15. Ahmed MM, Mubashir E, Wolf RE, Hayat S, Hall V, et al. (2006) Impact of treatment with infliximab on anticyclic citrullinated peptide antibody and rheumatoid factor in patients with rheumatoid arthritis. Southern Medical Journal 99: 1209-1215.

16. Morozzi G, Fabbroni M, Bellisai F, Cucini S, Simpatico A, et al. (2007) Low serum level of COMP, a cartilage turnover marker, predicts rapid and high ACR70 response to adalimumab therapy in rheumatoid arthritis. Clinical Rheumatology 26: 1335-1338.

17. Klaasen R, Thurlings RM, Wijbrandts CA, van Kuijk AW, Baeten D, et al. (2009) The relationship between synovial lymphocyte aggregates and the clinical response to infliximab in rheumatoid arthritis: a prospective study. Arthritis & Rheumatism 60: 3217-3224.

18. Bos WH, Bartelds GM, Vis M, van der Horst AR, Wolbink GJ, et al. (2009) Preferential decrease in IgG4 anti-citrullinated protein antibodies during treatment with tumour necrosis factor blocking agents in patients with rheumatoid arthritis. Annals of the Rheumatic Diseases 68: 558-563.

19. Dejaco C, Duftner C, Klotz W, Schirmer M, Herold M (2010) Third generation anti-cyclic citrullinated peptide antibodies do not predict anti-TNF-alpha treatment response in rheumatoid arthritis. Rheumatology International 30: 451-454.

20. Mu R, Huang H-Q, Li Y-H, Li C, Ye H, et al. (2010) Elevated serum interleukin 33 is associated with autoantibody production in patients with rheumatoid arthritis. Journal of Rheumatology 37: 2006-2013.

21. Onishi S, Yoshio T, Nagashima T, Minota S (2010) Decrease in the levels of anti-cyclic citrullinated peptide antibody in Japanese patients with rheumatoid arthritis who responded to anti-tumor necrosis factor-alpha. Modern Rheumatology 20: 528-530.

22. Charles PJ, Smeenk RJ, De Jong J, Feldmann M, Maini RN (2000) Assessment of antibodies to double-stranded DNA induced in rheumatoid arthritis patients following treatment with infliximab, a monoclonal antibody to tumor necrosis factor alpha: findings in open-label and randomized placebo-controlled trials. Arthritis & Rheumatism 43: 2383-2390.

23. Alessandri C, Bombardieri M, Papa N, Cinquini M, Magrini L, et al. (2004) Decrease of anti-cyclic citrullinated peptide antibodies and rheumatoid factor following anti-TNFalpha therapy (infliximab) in rheumatoid arthritis is associated with clinical improvement. Annals of the Rheumatic Diseases 63: 1218-1221.

24. Criswell LA, Lum RF, Turner KN, Woehl B, Zhu Y, et al. (2004) The influence of genetic variation in the HLA-DRB1 and LTA-TNF regions on the response to treatment of early rheumatoid arthritis with methotrexate or etanercept. Arthritis & Rheumatism. pp. 2750-2756.

25. Nissinen R, Leirisalo-Repo M, Peltomaa R, Palosuo T, Vaarala O (2004) Cytokine and chemokine receptor profile of peripheral blood mononuclear cells during treatment with infliximab in patients with active rheumatoid arthritis. Annals of the Rheumatic Diseases 63: 681-687.

26. Argyropoulou MI, Glatzouni A, Voulgari PV, Xydis VG, Nikas SN, et al. (2005) Magnetic resonance imaging quantification of hand synovitis in patients with rheumatoid arthritis treated with infliximab. Joint, Bone, Spine: Revue du Rhumatisme 72: 557-561.

27. Voulgari PV, Alamanos Y, Nikas SN, Bougias DV, Temekonidis TI, et al. (2005) Infliximab therapy in established rheumatoid arthritis: an observational study. American Journal of Medicine 118: 515-520.

28. Allaart CF, Goekoop-Ruiterman YPM, de Vries-Bouwstra JK, Breedveld FC, Dijkmans BAC, et al. (2006) Aiming at low disease activity in rheumatoid arthritis with initial combination therapy or initial monotherapy strategies: the BeSt study. Clinical & Experimental Rheumatology 24: S-77-82.

29. Parker A, Izmailova ES, Narang J, Badola S, Le T, et al. (2007) Peripheral blood expression of nuclear factor-kappab-regulated genes is associated with rheumatoid arthritis disease activity and responds differentially to anti-tumor necrosis factor-alpha versus methotrexate. Journal of Rheumatology 34: 1817-1822.

30. Scali JJ, Visentini S, Salomon J, Sevilla D, Ju YC, et al. (2007) Rapid and deep control of inflammation in rheumatoid arthritis with infliximab and its correlation with acute-phase reactants. Annals of the New York Academy of Sciences 1110: 389-401.

31. Yamanaka H, Tanaka Y, Sekiguchi N, Inoue E, Saito K, et al. (2007) Retrospective clinical study on the notable efficacy and related factors of infliximab therapy in a rheumatoid arthritis management group in Japan (RECONFIRM).[Erratum appears in Mod Rheumatol. 2007;17(2):178]. Modern Rheumatology 17: 28-32.

32. Tanaka Y, Takeuchi T, Inoue E, Saito K, Sekiguchi N, et al. (2008) Retrospective clinical study on the notable efficacy and related factors of infliximab therapy in a rheumatoid arthritis management group in Japan: one-year clinical outcomes (RECONFIRM-2). Modern Rheumatology 18: 146-152.

33. Canete JD, Suarez B, Hernandez MV, Sanmarti R, Rego I, et al. (2009) Influence of variants of Fc gamma receptors IIA and IIIA on the American College of Rheumatology and European League Against Rheumatism responses to anti-tumour necrosis factor alpha therapy in rheumatoid arthritis. Annals of the Rheumatic Diseases 68: 1547-1552.

34. Mattey DL, Brownfield A, Dawes PT (2009) Relationship between pack-year history of smoking and response to tumor necrosis factor antagonists in patients with rheumatoid arthritis. Journal of Rheumatology 36: 1180-1187.

35. Canete JD, Albaladejo C, Hernandez MV, Lainez B, Pinto JA, et al. (2011) Clinical significance of high levels of soluble tumour necrosis factor-alpha receptor-2 produced by alternative splicing in rheumatoid arthritis: a longitudinal prospective cohort study. Rheumatology 50: 721-728.

36. Di Muzio G, Perricone C, Ballanti E, Kroegler B, Greco E, et al. (2011) Complement system and rheumatoid arthritis: relationships with autoantibodies, serological, clinical features, and anti-TNF treatment. International Journal of Immunopathology & Pharmacology 24: 357-366.

37. Klarenbeek NB, van der Kooij SM, Guler-Yuksel M, van Groenendael JHLM, Han KH, et al. (2011) Discontinuing treatment in patients with rheumatoid arthritis in sustained clinical remission: exploratory analyses from the BeSt study. Annals of the Rheumatic Diseases 70: 315-319.

38. Curtis JR, Yang S, Chen L, Park GS, Bitman B, et al. (2012) Predicting low disease activity and remission using early treatment response to antitumour necrosis factor therapy in patients with rheumatoid arthritis: exploratory analyses from the TEMPO trial. Annals of the Rheumatic Diseases. pp. 206-212.

39. van Eijk IC, Nielen MMJ, van der Horst-Bruinsma I, Tijhuis GJ, Boers M, et al. (2012) Aggressive therapy in patients with early arthritis results in similar outcome compared with conventional care: the STREAM randomized trial. Rheumatology 51: 686-694.

40. Cavazzana I, Bobbio-Pallavicini F, Franceschini F, Bazzani C, Ceribelli A, et al. (2007) Anti-TNF-alpha treatment in rheumatoid arthritis with anti-Ro/SSA antibodies. Analysis of 17 cases among a cohort of 322 treated patients. Clinical & Experimental Rheumatology 25: 676-683.

41. Stojanovic S, Jevtovic-Stoimenov T, Stankovic A, Pavlovic D, Nedovic J, et al. (2011) Association of TNF-alpha Polymorphism (-308 A/G) with High Activity of Rheumatoid Arthritis and Therapy Response to Etanercept. Srpski Arhiv Za Celokupno Lekarstvo 139: 784-789.

42. Wang SY, Liu YY, Ye H, Guo JP, Li R, et al. (2011) Circulating Dickkopf-1 is correlated with bone erosion and inflammation in rheumatoid arthritis. The Journal of rheumatology. pp. 821-827.

43. Takeuchi T, Miyasaka N, Tatsuki Y, Yano T, Yoshinari T, et al. (2011) Baseline tumour necrosis factor alpha levels predict the necessity for dose escalation of infliximab therapy in patients with rheumatoid arthritis. Annals of the Rheumatic Diseases: 1208-1215.

44. Van Vollenhoven RF, Geborek P, Forslind K, Albertsson K, Ernestam S, et al. (2012) Conventional combination treatment versus biological treatment in methotrexate-refractory early rheumatoid arthritis: 2 Year follow-up of the randomised, non-blinded, parallel-group Swefot trial. The Lancet 379: 1712-1720.

45. Zivojinovic SM, Pejnovic NN, Sefik-Bukilica MN, Kovacevic LV, Soldatovic II, et al. (2012) Tumor necrosis factor blockade differentially affects innate inflammatory and Th17 cytokines in rheumatoid arthritis. Journal of Rheumatology 39: 18-21.

46. Lagana B, Picchianti Diamanti A, Ferlito C, Germano V, Migliore A, et al. (2009) Imaging progression despite clinical remission in early rheumatoid arthritis patients after etanercept interruption. International Journal of Immunopathology & Pharmacology 22: 447-454.

47. van der Kooij SM, Goekoop-Ruiterman YPM, de Vries-Bouwstra JK, Guler-Yuksel M, Zwinderman AH, et al. (2009) Drug-free remission, functioning and radiographic damage after 4 years of response-driven treatment in patients with recent-onset rheumatoid arthritis. Annals of the Rheumatic Diseases 68: 914-921.

48. Verstappen SMM, Lunt M, Bunn DK, Scott DGI, Symmons DPM (2011) In patients with early inflammatory polyarthritis, ACPA positivity, younger age and inefficacy of the first non-biological DMARD are predictors for receiving biological therapy: results from the Norfolk Arthritis Register. Annals of the Rheumatic Diseases 70: 1428-1432.
