# Supplementary material for: The Status of Rheumatoid Factor and Anti-Cyclic Citrullinated Peptide Antibody Are Not Associated with the Effect of Anti-TNFα Agent Treatment in Patients with Rheumatoid Arthritis: A Meta-Analysis
Source: PLoS One. 2014 Feb 27;9(2):e89442. doi: 10.1371/journal.pone.0089442 (PMC3937352; doi:10.1371/journal.pone.0089442)
Supplement: File S2 — PRISMA Checklist. (DOC) [file pone.0089442.s002.doc]

| **Section/topic** | **#** | **Checklist item** | **Section of paper** |
| --- | --- | --- | --- |
| **TITLE** | | |  |
| Title | 1 | The status of rheumatoid factor and anti-cyclic citrullinated peptide antibody are not associated with the effect of anti-TNFα agent treatment in patients with rheumatoid arthritis: A meta-analysis | Tile |
| **ABSTRACT** | | |  |
| Structured summary | 2 | Objectives. This meta-analysis was conducted to investigate whether the status of rheumatoid factor (RF) and anti-cyclic citrullinated peptide (anti-CCP) antibody are associated with the clinical response to anti-tumor necrosis factor (TNF) alpha treatment in rheumatoid arthritis (RA).  Methods. A systemic literature review was performed using the MEDLINE, SCOPUS, Cochrane Library, ISI Web of Knowledge, and Clinical Trials Register databases, and Hayden’s criteria of quality assessment for prognostic studies were used to evaluate all of the studies. The correlation between the RF and anti-CCP antibody status with the treatment effect of anti-TNFα agents was analyzed separately using the Mantel Haenszel method. A fixed-effects model was used when there was no significant heterogeneity; otherwise, a random-effects model was applied. Publication bias was assessed using Egger’s linear regression and a funnel plot.  Results. A total of 14 studies involving 5561 RA patients meeting the inclusion criteria were included. The overall analysis showed that the pooled relative risk for the predictive effects of the RF and anti-CCP antibody status on patient response to anti-TNFα agents was 0.98 (95% CI: 0.91-1.05, p=0.54) and 0.88 (95% CI: 0.76-1.03, p=0.11), respectively, with I2 values of 43% (p=0.05) and 67% (p<0.01), respectively. Subgroup analyses of different anti-TNFα treatments (infliximab vs. etanercept vs. adalimumab vs. golimumab), response criteria (DAS28 vs. ACR20 vs. EULAR response), follow-up period (≥ 6 vs. < 6 months), and ethnic group did not reveal a significant association for the status of RF and anti-CCP.  Conclusions. Neither the RF nor anti-CCP antibody status in RA patients is associated with a clinical response to anti-TNFα treatment. | Abstract |
| **INTRODUCTION** | | |  |
| Rationale | 3 | Both rheumatoid factor (RF) and antibodies against cyclic citrullinated peptides (anti-CCP) are regarded as serological markers of RA [5,6]. Some studies suggested that RF or anti-CCP antibody status in RA patients was associated with clinical response to anti-TNFα treatment [7-14], whereas in other studies this was not found [15-19]. And no definite conclusion has been reached up to now. (references presented in the article) | Introduction |
| Objectives | 4 | We carried out this meta-analysis to investigate whether RF and anti-CCP has predictive value for clinical response to anti-TNFα treatment. Studies working on association of status of RF or anti-CCP antibody and response to anti-TNFα treatment were searched and included in this meta-analysis. We also performed subgroup analysis on different variables in order to explore potential source of independent predictive factors for treatment effect of anti-TNFα treatment. | Introduction |
| **METHODS** | | |  |
| Protocol and registration | 5 | A literature search was performed for all studies that evaluating the association between RF or anti-CCP antibody status and response to anti-TNFα therapy in RA patients. | Methods |
| Eligibility criteria | 6 | A study was included if 1) patients were elder than 16 years old; diagnosed with RA using ACR criteria; and treated with at least one of the anti-TNFα agents (adalimumab, infliximab, etanercept, certolizumab, and golimumab); 2) efficacy was measured with EULAR or ACR or DAS28 criteria after minimum duration of 12 weeks; 3) the status of RF or anti-CCP antibody at baseline and sufficient data to calculate risk ratio (RR) were reported in the study. | Methods |
| Information sources | 7 | Databases included Medline, Cochrane library, SCOPUS (including EMbase), ISI web of knowledge and Clinical trial register(clinical trials.gov) . | Methods |
| Search | 8 | The following key words were searched: rheumatoid arthritis, anti-TNFα, rheumatoid factor, anti-cyclic citrullinated peptide antibody, clinical trials and systematic review. Synonym and spelling variation were taken into account. There was limitation of language but not year of publication. We only considered the publication in English. Citations were reviewed to search relevant original studies. And electronic search alert was set to cover recent studies. | Methods |
| Study selection | 9 | Three individual investigators (QL, YY & XL) evaluated the references respectively. Decision for inclusion was made on consensus. A study was included if 1) patients were elder than 16 years old; diagnosed with RA using ACR criteria; and treated with at least one of the anti-TNFα agents (adalimumab, infliximab, etanercept, certolizumab, and golimumab); 2) efficacy was measured with EULAR or ACR or DAS28 criteria after minimum duration of 12 weeks; 3) the status of RF or anti-CCP antibody at baseline and sufficient data to calculate risk ratio (RR) were reported in the study. | Methods |
| Data collection process | 10 | We collected the full text from internet if possible. We also contacted authors to request full text review or specific data of studies if there was no electronic version of the full text or sufficient data of studies needed for this meta-analysis. | Methods |
| Data items | 11 | **Funding:** This work was supported by grants from the National Natural Science Foundation of China (81172859, 81273312), National Science Fund for Distinguished Young Scholars (81325019), and the Capital Health Research and Development of Special (2011-4001-02).  Search studies: all studies that evaluating the association between RF or anti-CCP antibody status and response to anti-TNFα therapy in RA patients. | Methods, Funding |
| Risk of bias in individual studies | 12 | All studies included were evaluated for the potential bias by Hayden's criteria of quality assessment of prognostic studies . Six domains were considered in the bias assessment method, which were “study participation”, “study attrition”, “prognostic factor measurement”, “outcome measurement”, “confounding measurement and account”, and “analysis”. Scores “0”, “1”, “2” represented “high bias”, “partial bias” and “low bias” respectively. If available information was not enough to get a decision after contacting the authors, “unsure” was used and no score was calculated. And total scores were calculated to show the level of study quality. Studies with score at “11-12” were scaled as having relatively high quality while “9-10” as moderate and “less than 8” as low quality. Evaluation was performed by three investigators (QL, YY & XL) independently to improve the validity. | Methods |
| Summary measures | 13 | Point estimate of risk ratio (RR) and 95% confidence intervals (CI) were calculated. | Methods |
| Synthesis of results | 14 | Mantel Haenszel analysis was used to calculate the pooled RR and 95% CI. Heterogeneity of effects across studies was assessed using the Chi square test and qualified by I^2^ values, which represents the proportion of between-study variability attributable to heterogeneity rather than chance. I^2^ values of 25%, 50%, and 75% are referred to as low, moderate, and high heterogeneity effects, respectively. Fixed effects model was used when there was no significant heterogeneity. Otherwise, random effects model was used. | Methods |

Page 1 of 2

| **Section/topic** | **#** | **Checklist item** | **Section of paper** |
| --- | --- | --- | --- |
| Risk of bias across studies | 15 | Publication bias was estimated by funnel plots and Egger’s test, when P<0.05 was considered representing significant bias. | Methods |
| Additional analyses | 16 | Sensitive analysis was performed by sequential omission of individual studies.  Subgroup analysis was conducted to investigate the potential source of heterogeneity. It was performed by stratifying on different anti-TNFα agents, response criteria, duration of follow up, and ethnic group. | Methods |
| **RESULTS** | | |  |
| Study selection | 17 | All of 1649 records were identified for screening. 14 studies including 5561 patients were included. The other details are presented in Figure1 (flow diagram). | Results |
| Study characteristics | 18 | Data extracted from the 14 included studies were summarized in Table 1. | Results &Table 1 |
| Risk of bias within studies | 19 | Our assessment was performed on the basis of Hayden's criteria for prognostic studies. The result revealed that only one of the included studies was considered to be low quality, with a score of 7, while the other thirteen studies were considered as having moderate or high quality (11 moderate and 2 high) (Table 2). | Results & Table 2 |
| Results of individual studies | 20 | RR and 95% CI for each study are presented in Figure 2 and Figure 3. | See Figure2, Figure3 |
| Synthesis of results | 21 | The overall analysis of RF and anti-CCP antibody status showed the pooled RR were 0.98 (95% CI: 0.91-1.05, p=0.54) and 0.88(95% CI: 0.76-1.03, p=0.11), and I2 were 43% and 67%, showed in Figure 2, Figure 3 and Table 3. | Results |
| Risk of bias across studies | 22 | The funnel plot showed no significant evidence of asymmetry. And Egger’s linear regression test showed that the P values of RF and anti-CCP antibody status analysis were 0.777 and 0.422. | Results |
| Additional analysis | 23 | Sensitivity analyses were performed based on the results of the bias assessments, which was Hayden's criteria of quality assessment for prognostic studies, by sequential omission of individual studies. And the result revealed the significance estimate of overall pooled RR was not influenced by omitting any single study.  Subgroup analysis on different anti-TNFα agents, follow-up period, response criteria, and ethnic groups were performed to investigate the source of heterogeneity, and the results revealed no significant difference | Results |
| **DISCUSSION** | | |  |
| Summary of evidence | 24 | The analysis based on the data extracted from included 14 studies indicated that RF and anti-CCP antibody status were not associated with clinical response to anti-TNFα treatment in RA patients. This result suggests there is no enough evidence indicating predictive value of RF or anti-CCP antibody status in choosing anti-TNF α agents for RA patients. | Discussion |
| Limitations | 25 | 1. only 14 studies were included in this meta-analysis, with 12 studies reporting the status of RF and 10 studies reporting the status of anti-CCP antibody. 2. all the patients of included studies in our meta-analysis happened to have long-standing disease with the mean value of disease duration ranging from 5 to 14 years and had previously failed at least one kind of DMARDs. 3. most of the included studies used a combined treatment of DMARDs in a proportion of the patients enrolled. | Discussion |
| Conclusions | 26 | RF and anti-CCP antibody status were not associated with clinical response to anti-TNFα treatment in RA patients | Discussion |
| **FUNDING** | | |  |
| Funding | 27 | This work was supported by grants from the National Natural Science Foundation of China (81172859, 81273312), National Science Fund for Distinguished Young Scholars (81325019), and the Capital Health Research and Development of Special (2011-4001-02). | Funding |

*From:*  Moher D, Liberati A, Tetzlaff J, Altman DG, The PRISMA Group (2009). Preferred Reporting Items for Systematic Reviews and Meta-Analyses: The PRISMA Statement. PLoS Med 6(6): e1000097. doi:10.1371/journal.pmed1000097

For more information, visit: **www.prisma-statement.org**.

Page 2 of 2
